# Students’ Learning Outcomes in a Virtual Microscopic Online and Face-to-Face Anatomy Course: Cross-Over Comparative Study

**DOI:** 10.2196/82012

**Published:** 2026-09-16

**Authors:** Katharina Sternecker, Christoph Schmitz, Markus Berndt

**Affiliations:** 1 Department of Anatomy II, LMU Medizin, Ludwig-Maximilians-Universität München, Pettenkoferstr. 11, Munich, 80336, Germany, 49 89-2180-72704, 49 89-2180-72683; 2 Institute of Medical Education - LMU University Hospital, LMU Medizin, Ludwig-Maximilians-Universität München, Munich, Germany

**Keywords:** virtual slides, hybrid course, learning outcomes, demographics, blended learning

## Abstract

**Background:**

Since 2021, the histology of organs in the microscopic anatomy course at the Department of Anatomy II of the Ludwig-Maximilians-Universität Munich has been held as a hybrid course using a 3D virtual microscopy system. Evidence comparing synchronous online versus face-to-face instruction using an advanced 3D virtual slide system remains limited. In particular, it is unclear whether learning outcomes are equivalent across instructional modes, and whether individual student characteristics influence learning outcomes under these conditions.

**Objective:**

This study systematically (1) compares the learning outcomes of preclinic students attending a microscopic anatomy course either face-to-face or synchronously online using a 3D virtual microscopy slide box generated from fully scanned histological glass slides previously used in a traditional course, and (2) investigates whether selected demographic characteristics and personal skills predict learning outcomes in either instructional mode. We hypothesized that learning outcomes would not differ between online and face-to-face participation when instructional conditions are identical.

**Methods:**

The students attended 2 consecutive course days on 2 topics (kidney and liver) in a crossover design. Half of the students attended the first day face-to-face, and the second day online, while the other half attended in the reverse order. Demographic characteristics (eg, self-organization and affinity for technology interaction) and learning outcomes were collected via survey and simulated examination on the day after the second course day. To minimize potential confounding factors (eg, learning by means of external resources) and to ensure that the results reflected the learning outcomes within the scope of this study, 2 particularly challenging course days were intentionally selected, and the time interval before the simulated examination was kept short. Learning outcomes were analyzed using a linear mixed-effects model, and potential predictors were explored using stepwise linear regression.

**Results:**

Overall, 740 student questionnaires and examinations (740/864, 86% of the total student cohort) were used to assess the items' quality of the simulated examination. Inferential analyses included data from 415 students who completed the crossover design as intended. No significant main effect of participation mode on learning outcomes was found (*F*_1, 413_=0.16; *P*=.69). Although a significant effect of examination topic was observed (*F*_1, 413_=25.56; *P*<.001), there was no significant interaction between participation mode and examination topic (*F*_1, 413_=0.13; *P*=.72). Regression analyses showed low predictive power overall (*R*^2^≥0.12), with self-organization emerging as a weak but consistent predictor across both instructional modes.

**Conclusions:**

The results of this study suggest that the microscopic anatomy learning outcomes of students under controlled short-term conditions are comparable in synchronous online and face-to-face teaching. However, it should be noted that the study was limited to 2 topics and 2 consecutive course days. Further research should address additional topics, long-term learning outcomes, and broader student populations.

## Introduction

In recent years, 2 developments have taken place in Germany that were challenging for both learners and instructors at universities: (1) the continuous advancement of digital teaching and (2) the growing heterogeneity of students. Students at universities in Germany are becoming increasingly heterogeneous regarding demographic factors such as socioeconomic background, ethnic origin, financing of studies, economic situation, employment, housing situation, or personal and study-related factors such as prior educational background, digital literacy, time commitments (eg, part-time work and family responsibilities), study experience, and learning strategies [1-3]. There is a steadily growing demand for distance learning offerings at universities worldwide, particularly if there are in-person class locations that can only be reached with considerable time effort by students, for example, when locations are hard to access because of long travel distances or densely scheduled timetables [4-6]. This growing diversity poses substantial challenges for curriculum design, particularly in demanding and content-dense disciplines such as anatomy.

During the COVID-19 pandemic, medical schools worldwide switched to remote learning in the preclinical phase, and many subjects, including microscopic anatomy, were taught online [7]. As it could be shown that the online formats were well received by students and instructors, they were partially retained. Overall, constantly evolving technological possibilities have led to an increased use of virtual slides (ie, digital copies of glass slides) for teaching microscopic anatomy in the laboratory [8,9], and it has even been observed that virtual microscopy (VM) has a slight advantage over optical microscopy (OM) in teaching microscopic anatomy [10,11]. Presumably, this is due to the greater accessibility of the technology for students compared to conventional microscopy. Furthermore, the shared access to identical specimens supports collaborative learning among students. Virtual 2D microscopy is well accepted and widely used in microscopic anatomy courses, as it shows good results regarding implementation, relevance, and educational effectiveness of microscopic anatomy [12,13], but virtual 3D microscopy is generally not used because of the considerable effort required to produce virtual 3D slides. Moreover, online teaching is viewed with skepticism, as negative experiences regarding learning success in anatomy have been reported in online teaching of anatomy [14]. Beyond technical challenges, insufficient peer interaction and the limited use of online platforms for communication were identified. Holland et al [15] showed that students who attended fewer face-to-face classes than their peers were also less active in online offerings. In this study, student attendance was correlated with their learning success. The online offerings were supplementary to the face-to-face classes, meaning it was not examined whether students would have shown a greater willingness to participate in the lessons if the entire traditional face-to-face course had been offered online. This is important because time spent on microscopy of histological specimens is essential for the later transfer of knowledge [16].

It can be seen that in such far-reaching changes like switching from face-to-face to online teaching, oftentimes insufficient attention is directed towards the existence of specific prerequisites or personality traits of students that enable them to adapt to the new conditions effectively. For example, it is not clear whether online teaching is used equally by all students or can be used by all students, and how the individual skills and personal environment of students affect their learning success in online teaching. It has been demonstrated that the social environment outside the university and the personal abilities of students can influence their learning success and, consequently, their academic success [17]. So far, there is limited knowledge about which groups of students require additional learning opportunities in addition to the offered online teaching, as students may not have the same skills and/or comparable social or technical prerequisites. There is insufficient understanding of students whose learning success may improve through online teaching (eg, students who have to work alongside their studies to support themselves), so that they can be adequately supported through appropriate instruction. It has been found, for example, that academically weaker students are more affected by a reduction in the histology curriculum than stronger students [18]. Therefore, the question arises whether the instructional mode, online or face-to-face, influences learning success depending on sociodemographic characteristics and personal factors. In addition to that, existing studies investigated teaching in online courses or additional online offerings alongside the face-to-face course in microscopic anatomy by examining entire courses conducted over several months. While such designs provide valuable insights into overall course effectiveness, they also introduce numerous potential confounders, including prolonged self-study, intensive use of external learning resources, formation of peer learning groups, and cumulative assessment effects. As a result, it remains difficult to isolate the specific contribution of instructional modality (online vs face-to-face) to observed learning outcomes.

Theoretical frameworks such as aptitude-treatment interaction (ATI) studies emphasize that learning outcomes are not only determined by instructional methods, but by interactions between learner aptitudes and instructional conditions. ATI is related to complex patterns on its own but also depends on the desired learning content and sociodemographic characteristics [19]. In this context, it is generally assumed that weaker and anxious students are positively influenced in their learning success by a well-organized learning environment, whereas stronger, nonanxious students may be hindered by it. From this perspective, these studies focus on connections between learning success and sociodemographic characteristics or personal skills. In relation to hybrid classes, it is important to know whether any of the implemented formats advantage or disadvantage particular student groups and whether additional support mechanisms are required to ensure equitable learning opportunities.

At Ludwig-Maximilians-Universität München (Ludwig Maximilian University [LMU], Munich), since 2021, the course on microscopic anatomy has been conducted in a hybrid format (ie, face-to-face and synchronously streamed online participation options) from course days 13 to 26 of the microscopic anatomy course. This approach ensures that students who followed the online option received the same instructions as those attending face-to-face. Online students can communicate with the instructor and/or assistants via chat or audio; students attending face-to-face can talk to the instructors directly. However, it remained unclear whether the students’ learning outcomes in the online mode are comparable to face-to-face participation in the microscopic anatomy course using a virtual 3D slide box and whether individual demographic characteristics or personal skills meaningfully predict learning outcomes in either modality.

To address this gap, the aim of this study was (1) to investigate the learning outcomes of the students comparing the online participation with face-to-face attendance using Histologi@ as a virtual 3D slide box in microscopic anatomy and (2) to identify demographic characteristics and personal skills (eg, affinity for technology interaction) that may be able to predict the learning outcomes of medical students attending the microscopic anatomy course face-to-face or online. By focusing on a short, well-defined instructional period to control for confounding factors and by explicitly examining demographic characteristics and personal skills within an ATI framework, this study aimed to contribute robust empirical evidence to the ongoing discussion about the role of online and hybrid teaching in microscopic anatomy education.

## Methods

### Study Design

The study took place at the end of the microscopic anatomy course in the study year 2023/2024 at the Department of Anatomy of LMU Munich and consisted of 2 course days (28 and 29) and one day for data collection. The course days were spread over several days; the data collection took place in one day (Table 1).

**Table 1.** Cross-over design of this study: Class A-F represents the 6 classes of the students’ cohort taking the microscopic anatomy course during data collection; the students participated on 2 course days with different topics: course day 28 (topic 1=kidney) and course day 29 (topic 2=liver).

Course day and participation mode	Class A	Class B	Class C	Class D	Class E	Class F
Course day 28 (online)	A^a^	B^b^	A	B	A	B
Course day 28 (face-to-face)	B	A	B	A	B	A
Course day 29 (online)	B	A	B	A	B	A
Course day 29 (face-to-face)	A	B	A	B	A	B

a A: the students participated online first and then face-to-face afterwards.

b B: the students participated face-to-face first and then online afterwards.

The students in each class were divided into 2 groups. On course day 28, one group (Group A) participated online, while the other group (Group B) attended face-to-face. On course day 29, the groups switched formats. Group A and Group B were always instructed synchronously. Since the student cohort was split up to 6 classes, the course days 28 and 29, as well as the data collection (evaluation and simulated examination), were repeated 6 times.

Course days 28 (topic 1: kidney) and 29 (topic 2: liver) were conducted by trained instructors (one instructor per course day) and 2 to 3 additional teaching staff members per online or face-to-face condition, using the sandwich model to ensure well-defined teaching [20]. In the sandwich model, short interactive learning phases (active elements) are embedded between classic lecture segments to enhance attention and learning effectiveness. The content of each section is precisely scheduled with specified time allocations. In 10 sessions of the 2 course days, the first author served as the instructor, while in 2 sessions, the lead was taken by 2 instructors with experience in teaching microscopic anatomy. All instructors received a detailed briefing on the sandwich model from KS and used the same course materials on the course days.

The course days were held in the microscopic laboratories but were broadcast via Zoom (Zoom Communications, Inc). Consequently, the students received identical instructions, regardless of the type of participation. The class allocation was adopted from the dean’s office and was not modified for the study. Students independently chose in isolated cases to participate in a different group. Therefore, class attendance and the mode of participation were additionally assessed in the evaluation.

### Data Collection

The data collection took place face-to-face one day after course day 29 in the microscopic laboratories, and the instructor, as well as teaching staff members, monitored the data collection (course day 28 took place 3 or 4 days before course day 29, depending on the schedule). The students were not kept from independently using the Histologi@ system or engaging with the course materials in the period between the course days and the simulated examination and the evaluation, as data collection took place during the regular semester.

The evaluation survey and examination were conducted using EvaSys (evasys GmbH), and the students could participate digitally via a QR code. On average, students took about 7.5 (SD 0.4) min to complete the evaluation, immediately followed by a 30-minute time slot to answer the examination questions.

### Simulated Examination and Instruments

To each single-choice question (5 answer options), a carefully selected 2D image section from the virtual 3D slides shown in both course days was assigned and projected onto the board for one minute. During that one minute, the students were instructed to submit their response digitally via EvaSys. In order for the students to answer the question correctly, the students first had to identify the image, and then answer a question related to that specific image section. The students were not informed in advance which image sections from the virtual 3D slides would be used for the simulated examination.

The simulated examination consisted of 30 single-choice questions, 15 referring to course day 28 (kidney) and 15 referring to course day 29 (liver). The questions corresponded to the standard of the final examination for the course in microscopic anatomy at the Department of Anatomy. All examination items were developed by subject matter experts through group consensus.

The evaluation assessed demographic characteristics (3 items on age, commuting time, and working hours to finance studies), learning preferences (7 items on, eg, self-organization, preference for practical work, and attitudes toward group work), and affinity for technology interaction (9 items), all on a 5-point Likert scale (1=not correct at all; 5=completely correct). Detailed evaluation items are provided in Table S1 in Multimedia Appendix 1.

Commuting time was considered particularly relevant because medical education at LMU does not take place on a centralized campus; instead, students often have to travel between different teaching locations and may spend several hours per day commuting in order to attend various classes. As digital tools are playing an ever-growing role in teaching, and students’ technical affinity may influence their learning outcomes, and as online participation with virtual microscopy may therefore present a challenge, this item was included as well. The affinity for technology interaction was calculated according to Franke et al [21].

Only fully completed evaluations were included in the analysis. The survey items were reviewed by the ethics committee and revised where necessary.

### Microscopic Anatomy Course at the Department of Anatomy at LMU Munich

864 students attended the microscopic anatomy course in the study year 2023/2024. Due to the high number of students, the cohort was split up into 6 classes (A-F), each with approximately 144 students. The allocation of the students into the classes was carried out randomly at the beginning of the study year by the dean’s office of studies. Sixty-eight workstations in the main microscopic anatomy laboratory and 21 workstations in a second smaller microscopic anatomy laboratory were provided at the Department of Anatomy for face-to-face participation in the microscopic anatomy course for students who wanted to attend the microscopic anatomy course face-to-face. At each workstation, 1‐2 students can use the virtual 3D slide box of Histologi@ with a Mac-computer (M1 Mac and iMac, Apple Inc) with macOS High Sierra or macOS Monterey (Apple Inc) for virtual 3D microscopy.

The microscopic anatomy course at the Department of Anatomy of the LMU Munich consists of 29 course days and includes cell biology and tissue science (course days 1‐15), as well as the histology of the organs (course days 16‐29). The students attended the course days 1‐15 exclusively in face-to-face mode. From course day 15 until course day 29, the course was conducted in a hybrid format (ie, face-to-face and online). For students who wished to participate online, the face-to-face classes were streamed synchronously via Zoom from the main microscopic laboratory, and they could use Histologi@ with Biolucida Viewer (MBF Bioscience) on their own personal devices. This approach ensured that students who chose the online mode received the same instruction as those attending face-to-face. Online students had the option to communicate with the instructor and/or additional teaching staff members via chat or audio, which allows the teaching staff an interactive supervision as is common in the microscopic anatomy laboratory.

### Virtual Microscopy System

The students have access to the virtual 3D slide box of Histologi@ via MBF server software, which reads the 3D-files and compiles the data stream necessary for the visualization in the Biolucida Cloud Viewer 2020.1.0 (MBF Bioscience) [22].

Via cloud (Leibniz-Rechenzentrum der Bayerischen Akademie der Wissenschaften, Garching, Germany), students can access Histologi@ not only at the workstations, but also with their own personal devices, and they study the virtual 3D slide box location-independently.

The students used Histologi@ to learn microscopic anatomy by studying 197 virtual 3D slides. A distinctive feature of the program is that the glass slide collection was digitized with a research-grade microscope, including not only the x- and y-axis information but also the z-axis (ie, several overlying focus planes of the same slide), resulting in file sizes of the individual 3D virtual slides. Therefore, students can directly magnify the virtual slides and change the focus plane, as was previously common practice with analog microscopy.

All 3D virtual slides of Histologi@ can be obtained for free from the authors of this study. The content-related use of the virtual slides and the theoretical content of the course is summarized in lecture notes available free of charge and in a book [23,24].

### Ethical Considerations

The study was reviewed and approved by the Ethics Committee of LMU Munich, Munich, Germany (Project reference number 22‐0152 KB, February 14, 2022). The committee confirmed that the study raised no ethical or legal concerns in accordance with the Declaration of Helsinki, § 15 of the Professional Code for Physicians in Bavaria, and applicable faculty regulations.

Data collection for both the evaluation and the examination was blinded and entirely anonymous, with no personal identifiers recorded, thereby precluding any possibility of tracing responses to individual participants or influencing course completion outcomes. However, students readily accepted participation in the study as a means to support the creation of scientific evidence for future teaching formats and as a possibility to receive formative feedback on their learning progress. They had to confirm this through their active consent in the survey prior to the start of data collection. The students did not receive any monetary or other form of compensation for their participation.

### Sample Characteristics and Data Inclusion

**Figure 1.**
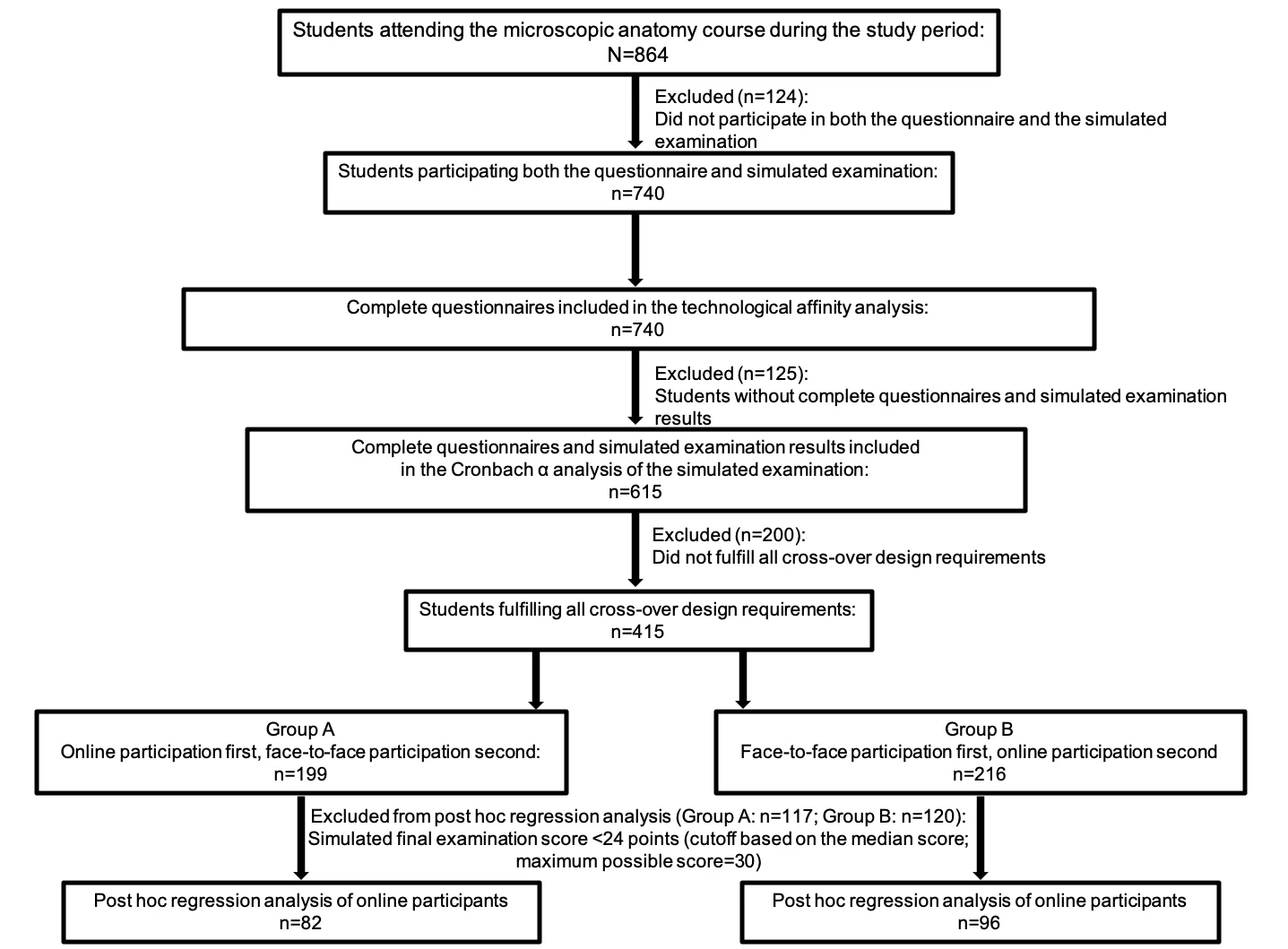
Flowchart of participant inclusion and analysis.

Only questionnaires were used in which the respective sections were fully completed. A total of 740 students completed both the questionnaire and the simulated examination, corresponding to approximately 86% (740/864) of the total cohort. Of these, 415 students fulfilled all requirements of the cross-over design and were included in the inferential statistical analysis. The selection of data included in the final analyses is illustrated in Figure 1.

Among the analyzed sample, 199 students (Group A) participated online first and face-to-face second, while 216 students (Group B) followed the reverse order.

Internal consistencies for the examination (30 items) and the technological affinity (9 items) were calculated using Cronbach α.

To calculate Cronbach α for the simulated examination, 615 questionnaires were included. For the calculation of the level of technological affinity, 740 questionnaires were used.

### Statistical Analysis

All calculations and graphics were performed using SPSS (IBM Corp). Cronbach α on testing the questions 1‐15 (topic 1=kidney) was 0.75, on testing the questions 16‐30 it was 0.75 (topic 2=liver), and overall (questions 1‐30) 0.85 (n=615). Therefore, all questions of the examination to evaluate the learning outcomes and all items to determine the level of technological affinity were used in the analysis.

Cronbach α on testing the 9 items to determine the level of technological affinity was 0.87 (n=740), signifying sufficient internal consistency.

Influence of the demographic characters and the personal skills were calculated using linear stepwise regressions. The stepwise regression analysis to predict the influence of demographic characteristics or personal skills was performed at four different levels: (1) across all learning outcomes; (2) across the learning outcomes for online and face-to-face participation on both course days; (3) across the learning outcomes of Group A, online and face-to-face participation, separately; and (4) across the learning outcomes of Group B, online and face-to-face participation, separately.

Additionally, a post hoc analysis of the online participants subgroup was conducted to allow for better identification of potential factors associated with lower outcomes after online instructions in greater detail. This analysis was considered exploratory and was performed to ensure that potential associations between demographic characteristics, personal skills, and learning outcomes in students who achieved lower scores in the simulated final examination after online instruction were not missed.

A cutoff of <24 points was applied because it corresponds to the median score achieved in the simulated final examination (maximum possible score=30). This approach was selected to differentiate between lower- and higher-performing students, as the median represents an objective cutoff and ensures balanced subgroup sizes.

Furthermore, separate regressions were conducted for this subgroup for course day 28 (n=82) and course day 29 (n=96), respectively. The comparison between the learning outcomes of online and face-to-face participation was analyzed using a linear mixed-effects model with restricted maximum likelihood estimation and an unstructured covariance matrix. Examination results were specified as the dependent variable. Participation mode (online vs face-to-face), course topic (liver vs kidney), and their interaction were included as fixed effects. The individual student was specified as the subject variable, and course topic day was modeled as the repeated factor.

## Results

### Effect of Participation Mode and Examination Topic

**Figure 2.**
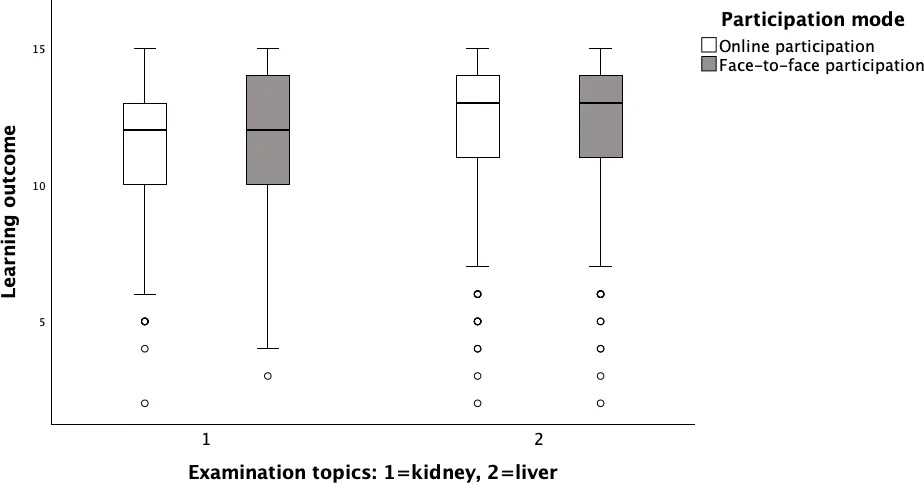
Box plots of medical students’ learning outcomes after online or face-to-face participation on course day 28 (kidney; online: n=199, face-to-face: n=216) and course day 29 (liver; online: n=216, face-to-face: n=199). Learning outcomes differed significantly between the 2 topics (linear mixed-effects model with an unstructured covariance matrix: *F*_1, 413_=25.56; *P*<.001); points indicate outliers; whiskers represent IQR.

The linear mixed-effects model, including participation mode (online vs face-to-face) and course topic (liver vs. kidney) as fixed effects and examination results as dependent variable, revealed no significant main effect of participation mode (*F*_1, 413_=0.16; *P*=.69) and a significant main effect of the examination topic (*F*_1, 413_=25.56; *P*<.001). No significant interaction between participation mode and examination topic was found (*F*_1, 413_=0.13; *P*=.72; Figure 2).

The means and SDs for the learning outcomes were 11.4 (SD 2.7) for course day 28 and 11.9 (SD 2.6) for course day 29. When comparing learning outcomes for online and face-to-face participation, the mean values were 11.6 (SD 2.7) for online and 11.7 (SD 2.6) for face-to-face participation.

### Predictors of Learning Outcomes

The model with the highest predictive probability was at level 4 (Table 2): the model in which the data from course day 28 of the online participants were used achieved a predictive probability of 12% with 3 predictors (commuting time, self-organization, and working hours per week; *P*<.001). The predictive probability was lower in the 3 other models at this level: for course day 28 with in-person participants, the predictive probability was 4% (*P*<.001); for course day 29 with online participants, it was 9% (*P*<.001); and for course day 29 with in-person participants, it was 7% (*P*<.001). In all 3 models, self-organization was also a predictor. For Group A (n=199), working hours per week was an additional predictor. Only in the model referring to course day 29 with online participants was intensive reviewing and reflective learning also identified as a predictor.

In every calculated model on the 4 levels, the ability for self-organization gave an indication of how great the learning outcomes can be, but the prediction probability for the model was low (detailed results of the models at 4 different levels are provided in Table S2 in Multimedia Appendix 2). It is remarkable that the influence was present regardless of whether the students participated online or face to face. Other personal skills that also had an influence on the prediction probability of the learning outcomes of the students could not be assigned to a participation mode. Students living nearer to the Department of Anatomy seem to have an advantage regarding the learning outcomes, regardless of the participation mode, as indicated by the results of the linear regressions analysis that were conducted. Commuting time as a predictor was identified in models that considered online participation as well as in those that considered face-to-face participation (Table S2 in Multimedia Appendix 2). However, the influence was minor for all the variables.

The analysis of the subgroup of low-performing participants showed that when only the results for online participation were considered, the model yielded an *R*² of 0.15 (*P*<.001; B=9.47, SE 0.84; 2-tailed *t*_174_=11.26). The significant predictors were self-organization (higher the value, better the performance; B=0.75, SE 0.18; *β*=.30; *t*_174_=4.19; *P*<.001), commuting time (longer the commute time, worse the performance; B=−0.03, SE 0.01; *β*=−.25; *t*_174_=−3.48; *P*<.001), and agreement with the statement “I prefer intensive reviewing and reflecting for learning” (lower the agreement, higher the scores; B=−0.47, SE 0.17; *β*=−.20; *t*_174_=−2.82; *P*=.005).

**Table 2.** Linear stepwise regression models to predict the learning outcomes: the model with the best predictability for the subgroup is shown. (1)=“I am very well self-organized when it comes to learning”; (2)=“What is your average travel time in minutes to the Anatomical Institute?”; (3)=“How old are you?”; (4)=“I prefer intensive reviewing and reflecting for learning.”; (5)=“I prefer practical tasks for learning” (6)=“How many hours per week do you work in addition to your studies to support yourself?”

Course days, participation mode, and predictor	B^a^	SE	β^b^	*t* test (*df*)	*P* value	*R* ^*2*^
Course day 28						
Online (n=199)						
—^c^	10.99	0.75	—	14.71 (195)	<.001	—
(2)	−0.04	0.01	−0.25	−3.53 (195)	<.001	0.12
(1)	0.48	0.18	0.18	2.70 (195)	.008	—
(6)	−0.06	0.03	−0.14	−2.01 (195)	.046	—
Face-to-face (n=216)						
—	9.46	0.65		14.65 (214)	<.001	—
(1)	0.50	0.16	0.20	3.05 (214)	.003	0.04
Course day 29						
Online (n=216)						
—	10.54	0.78	—	13.57 (213)	<.001	—
(1)	0.76	0.17	0.31	4.54 (213)	<.001	0.10
(4)	−0.43	0.16	−0.18	-2.65 (213)	.009	—
Face-to-face (n=199)						
—	10.40	0.68		15.29 (196)	<.001	—
(6)	−0.08	0.03	−0.20	−2.90 (196)	.004	0.07
(1)	0.49	0.17	0.20	2.86 (196)	.005	—

a B: unstandardized coefficient.

b *β*: standardized coefficient.

c Not applicable.

For students who attended only course day 28 online (model-yielded *R*²=.06; *P*<.001; B=9.75, SE 0.47; *t*_80_=20.73), commuting time remained the only significant predictor (B=−0.02, SE 0.01; *β*=−.24; *t*_80_=−2.24; *P*=.03).

For students who attended only course day 29 online, self-organization (B=1.52, SE 0.25, *β*=.46; *t*_91_=4.95; *P*<.001) and agreement with the statement “I prefer intensive reviewing and reflecting for learning” (B=−0.72, SE 0.22; *β*=−.29; *t*_91_=−3.25; *P*=.002) were also significant (again negatively correlated), and agreement with the statement “I prefer practical tasks for learning” (B=0.62, SE 0.26; *β*=.22; *t*_91_=2.44; *P*=.02) and commuting time (B=−0.03, SE 0.01; *β*=−.20; *t*_91_=−2.20; *P*=.03) additionally emerged as significant positive predictors. The model explained more variance in this group (*R*²=.28; *P*<.001; B=6.43, SE 1.69; *t*_91_=3.81).

Technological affinity was retained as a predictor in the linear regression models, because no association was detected between students’ technological affinity and their learning outcomes.

## Discussion

### Principal Findings

This study shows that there is no difference in the learning outcomes of the students using a virtual 3D slide box (Histologi@) and participating in the 2 anatomy course days face-to-face vs students using Histologi@ and participating in the course online via a common broadcast system.

### Interpretation of the Topic Effect

The difference in learning outcomes between the 2 course days may be due to the fact that the course day on which students achieved higher learning success was closer to the simulated examination.

This assumption was supported by the finding that, in the analysis of students with lower outcomes who attended course day 29 online, agreement with the statement “I prefer intensive reviewing and reflecting for learning” emerged as a significant predictor with a negative correlation. This was not the case for course day 28, which had taken place earlier. This suggests that, for the students of this subgroup, having more time for reflection and self-directed learning may have led to better results in this instance. To verify this assumption, the learning outcome would have needed to be reassessed at a later point in time.

The 2 topics, liver and kidney, were selected to ensure a content scope that was comparable. However, it is conceivable that differences in conceptual complexity between the 2 topics led to students performing better on the second topic, “liver.” For the purpose of addressing the research question, it is important that there was no significant difference between the instructional modes. Both groups of students, those who participated online on the respective day and those who attended in person, had similar learning outcomes.

### Individual Characteristics and ATI Framework

In the study, neither individual demographic characteristics nor individual skill variables were identified as significant predictors with high predictive power of the outcomes. The regression analyses showed that there were no prominent variables referring to demographic characteristics or personal skills analyzed in this study that had a significant influence on the learning outcomes in either condition, online versus face-to-face. The study clearly shows that the learning preferences (auditory, visual, or kinesthetic) that were surveyed by the questions of the learning preferences are not related to the prediction of the learning outcomes.

This may be partly attributable to the fact that the students had already been exposed to both online and face-to-face learning formats for an extended period of time. Moreover, they had been able to practice using the digital 3D system online for several months prior to the study.

Because the students were already familiar with the Histologi@ technical system and had the opportunity to practice with it, it is possible that eg, the learner characteristic of technological affinity was no longer sufficiently differentiated within the framework of ATI studies, and therefore the different instructional methods did not lead to significant effects. This suggests that it would be necessary to examine beginners who have not yet had the opportunity to practice with the system. In such a group, larger effects might become observable.

ATI-studies assume that certain environmental characteristics or personal abilities influence a person’s learning success in a specific learning environment [19]. ATI theory could be helpful at this point in guiding the development of personalized online interventions that support beginners in becoming familiar with the system. For example, within the ATI framework, it could also be investigated how long students with different aptitudes require to effectively engage with and follow the instructions online.

Another reason for the lack of detected differences may also be due to the fact that admission to medical school is subject to very strict selection criteria. For example, the final grade in the Abitur (German university entrance qualification) still plays a major role, as do the results in other qualifications that are relevant for admission to medical studies.

However, some variables can predict the learning outcome in microscopic anatomy to a small extent (eg, the assessment of self-organization). Since the probability of prediction is so small and especially since the variables make no general differences between the participation modes, it suggests that no major measures are necessary to adjust future online teaching accordingly to improve the learning conditions of individual groups with special characteristics tested in this study.

It is well known that self-organization, which includes self-regulated learning, is consistently associated with learning outcomes among medical students in particular [25]. Its predictive power was relatively modest in our study, and self-regulation did not differentiate between instructional modes.

Since self-regulation plays an especially important role in academic success during the clinical years, it should already be fostered in the preclinical phase, eg, by promoting autonomous learning structures or adapting the learning environment accordingly. Since, in our study, technical affinity did not emerge as a factor that led to a deterioration of students’ learning outcomes in the online mode, this may represent an opportunity to foster an environment that supports self-organization. Naturally, students’ level of experience must be considered for all measures taken.

### Comparison With Prior Work

The results of this study indicate that the decision to implement the possibility of online participation with Histologi@ via broadcast system could provide a good alternative for students in the microscopic anatomy course. Participation in the microscopic anatomy course with Histologi@, no matter whether online or face-to-face, can be equally effective for students’ learning progress, and for students of all levels of technological affinity. The use of Histologi@ under controlled short-term conditions is just as effective for medical students with their personal devices as it is in the microscopic laboratory at the provided workstations. Thus, our results confirm evidence that instruction in microscopic anatomy using virtual microscopy can be as effective as face-to-face instruction [26,27]. A distinctive feature of our study is that, within a clearly defined setting (comprising 2 topics covered over 2 course days followed by an immediate simulation examination) and compared with long-term studies a high control over external factors (eg, external study resources and group learning effects), we were able to show in a cross-over-designed study that learning outcomes did not differ between students attending online and those attending face-to-face. Previous studies compared the results of microscopic anatomy courses that were conducted over several months [26]. During this extended period, it is common for students to additionally study the course content using other resources (eg, asynchronous digital learning materials) and in learning groups.

The translation from a traditional microscopic anatomy course to VM was well planned and carefully prepared, both in terms of technology and in terms of having experienced or trained educators, as this is crucial for a successful transformation [28-30]. Short-term shifts from traditional analog to online VM, as it has oftentimes been the case during the COVID-19 pandemic, may not always yield the same outcomes, especially in subjects like anatomy, which rely on describing and understanding 3D structures [31,32]. In contrast to many COVID-era studies investigating emergency remote instruction, our study examined structured instructional modes under controlled conditions and did not have to be introduced abruptly due to a political decision. It had already been implemented over several course cycles, allowing instructors to become more familiar with face-to-face and online synchronous instructions than was the case during the COVID-19 pandemic. Students were not required to participate online but did so voluntarily. It can therefore be assumed that psychosocial stress, which also plays a role in academic performance, was greater during the COVID-19 pandemic.

### Relation to Broader Instructional Strategies

The curriculum for medical studies must be adapted to the changing requirements of medical education, ensuring the appropriate knowledge transfer to the students [18,33]. For example, the content of microscopic anatomy is increasingly integrated with clinical subjects. In order for students to engage with the study of microscopic anatomy regardless of the location of a microscopic laboratory, online instruction using VM is a good alternative to traditional face-to-face teaching. Being location-independent in teaching microscopic anatomy facilitates cooperation with clinical subjects, a key trend for future curriculum development [34,35]. Special consideration should be given to equipment and the learning environment in online teaching [36-38], and it should be carefully planned and not just be seen as a quick fix.

### Educational Implications

Although online teaching resulted in the same learning outcomes as face-to-face instruction in our study, one should not assume that this would automatically be true for all types of anatomy teaching. Other important factors in medical training that may not be experienced during online teaching have to be trained face-to-face, a teaching mode that is still deemed necessary by students for a profound medical education [39].

The Sandwich Model [20], for example, alternates between collective teaching phases and individual or small-group active learning phases, thereby maintaining attention and promoting cognitive processing. Structured Sequence Models, Team-based learning emphasizes structured collaboration, accountability, and application of knowledge in teams, while the Flipped Classroom model shifts content acquisition to preclass preparation and uses classroom time for higher-order application tasks and is also applied in preclinical anatomy education [40-42].

In contrast to these pedagogical approaches, which primarily focus on restructuring in-class interaction and collaborative learning, this study did not investigate a specific active-learning intervention but rather examined instructional modes within an established Sandwich Model framework. Thus, the study’s primary focus was not on comparing distinct didactic strategies but on evaluating learning outcomes across instructional delivery formats. However, it should also be reconsidered and further investigated to what extent instructional formats such as Problem-based learning or Team-based learning, particularly when implemented in an online setting, are suitable for the acquisition of foundational knowledge in the preclinical phase. In addition to teaching formats, diverse learning styles should be considered because it can also increase the effectiveness of learning units and should be examined in future studies [43].

### Strengths and Limitations of the Study

A high number of participants was reached, and the study design was implemented under controlled circumstances, resulting in high statistical power.

To restrict external influences such as courses and examinations in other subjects or extended holiday breaks, the study was limited to 2 course days. In addition, efforts were made to minimize disruption to the regular teaching schedule.

For the study, the organization and the traditional structure of the microscopic anatomy course had to be adjusted. Timely information and transparent communication (eg, via email or presentation) were offered to minimize reservations from instructors, organizers, and students.

The students already had several weeks of experience applying histology in the histology laboratory when the study took place at the end of the course. Therefore, the study does not necessarily apply to students without experience in the use of virtual microscopy.

An important limitation of this study is that, although the students were encouraged to attend either the face-to-face or the online session on their assigned course day, due to the predefined framework conditions under which the study was conducted, the students ultimately had the option to participate online on their preferred day. As a result, a potential bias due to carry-over effects cannot be excluded. Nevertheless, since the students had already been working with the program for several weeks prior to this study, it can be assumed that the self-selected order of online and face-to-face participation did not introduce substantial bias. However, in future studies, it would be advisable to enforce a strictly randomized allocation of the participation mode and its sequence.

Additionally, potential confounding variables such as academic performance, motivation, and external study time were not examined in this study. These variables may also have influenced the learning outcomes.

### Conclusions

With careful planning and the right equipment, online teaching in microscopic anatomy using virtual 3D slides can be comparable to face-to-face teaching in terms of digital microscopy trained student learning outcomes. The medical students at the Ludwig-Maximilians-Universität bring the necessary prerequisites with them at the end of the microscopic anatomy course to achieve similar learning outcomes in both online and face-to-face instruction using Histologi@. This suggests that students are able to follow the content of digital instruction in microscopic anatomy as effectively as face-to-face instruction. However, it should also be recognized that students do not always have the opportunity to gain extensive experience with virtual microscopy in a face-to-face setting before transitioning to online formats. Investigations of digital teaching formats involving novice students may help to better differentiate associations between demographic characteristics and personal skills and learning outcomes. In further studies on online teaching, different instructional formats and learning preferences should also be considered to determine whether these factors may influence learning outcomes differently across instructional modes.

## Supplementary material

10.2196/82012 Multimedia Appendix 1 Items of the evaluation that related to demographic characteristics of diversity and personal skills were shown.

10.2196/82012 Multimedia Appendix 2 Linear stepwise regression models to predict the learning outcomes: The model with the best predictability for the subgroup is shown.

## Abbreviations

ATI: aptitude-treatment interaction
LMU: Ludwig Maximilian University
OM: optical microscopy
VM: virtual microscopy
