# Supplementary material for: Students’ Learning Outcomes in a Virtual Microscopic Online and Face-to-Face Anatomy Course: Cross-Over Comparative Study
Source: JMIR Med Educ. 2026 Sep 16;12:e82012. doi: 10.2196/82012 (PMC13580892; doi:10.2196/82012)
Supplement: Supplementary file 1 [file mededu-v12-e82012-s001.docx]

**Multimedia Appendix 1**

Items of the evaluation that related to demographic characteristics of diversity and personal skills were shown.

| **Item categories** | **Item** |
| --- | --- |
| *a) Items referring to demographic characteristics:* | i. How old are you? |
|  | ii. What is your average travel time in minutes to the Anatomical Institute? |
|  | iii. How many hours per week do you work in addition to your studies to support yourself? |
| *b) Items referring to learning preferences (Likert scale 1 (not correct at all) - 5 (completely correct):* | i. I prefer oral explanations for learning. |
|  | ii. I prefer a language rich in gestures and images for learning, as used by the instructor. |
|  | iii. I prefer practical tasks for learning. |
|  | iv. I prefer intensive reviewing and reflecting for learning. |
|  | v. I prefer intensive conversations and discussions for learning. |
|  | vi. I am very well self-organized when it comes to learning. |
|  | vii. In online classes, it is just as easy for me to join a study group if needed, as it is in face-to-face classes. |
| *c) Items referring to affinity for technology interaction (Likert scale 1 (not correct at all) - 5 (completely correct):* | i. I like to occupy myself in greater detail with technical systems. |
|  | ii. I like testing the functions of new technical systems |
|  | iii. I predominantly deal with technical systems because I have to. |
|  | iv. When I have a new technical system in front of me, I try it out intensively. |
|  | v. I enjoy spending time becoming acquainted with a new technical system. |
|  | vi. It is enough for me that a technical system works; I don’t care how or why. |
|  | vii. I try to understand how a technical system exactly works. |
|  | viii. It is enough for me to know the basic functions of a technical system. |
|  | ix. I try to make full use of the capabilities of a technical system. |
