# Supplementary material for: Students’ Learning Outcomes in a Virtual Microscopic Online and Face-to-Face Anatomy Course: Cross-Over Comparative Study
Source: JMIR Med Educ. 2026 Sep 16;12:e82012. doi: 10.2196/82012 (PMC13580892; doi:10.2196/82012)
Supplement: Supplementary file 2 [file mededu-v12-e82012-s002.docx]

**Multimedia Appendix 2**

Linear stepwise regression models to predict the learning outcomes: The model with the best predictability for the subgroup is shown.

|  | **n** | **Participation mode** | **Predictor** | **B** | **SE B** | **β** | **t** | **p** | **R^2^** |
| --- | --- | --- | --- | --- | --- | --- | --- | --- | --- |
| Both course days | 830 | online and face-to-face | Intercept | 12.302 | 0.827 |  | 14.876 | <.001 |  |
|  |  |  | 1) | 0.612 | 0.088 | 0.242 | 6.972 | <.001 | 0.089 |
|  |  |  | 2) | -0.019 | 0.005 | -0.129 | -3.817 | <.001 |  |
|  |  |  | 3) | -0.107 | 0.033 | -0.111 | -3.290 | .001 |  |
|  |  |  | 4) | -0.253 | 0.085 | -0.104 | -2.957 | .003 |  |
|  |  |  | 5) | 0.198 | 0.095 | 0.070 | 2.085 | .037 |  |
| Both course days | 415 | online |  | 11.213 | 0.603 |  | 18.608 | <.001 |  |
|  |  |  | 1) | 0.637 | 0.125 | 0.248 | 5.090 | <.001 | 0.101 |
|  |  |  | 2) | -0.023 | 0.007 | -0.152 | -3.180 | .002 |  |
|  |  |  | 6) | -0.059 | 0.023 | -0.121 | -2.547 | .011 |  |
|  |  |  | 4) | -0.284 | 0.120 | -0.115 | -2.359 | .019 |  |
|  | 415 | face-to-face |  | 12.647 | 1.023 |  | 12.365 | <.001 |  |
|  |  |  | 1) | 0.509 | 0.119 | 0.205 | 4.288 | <.001 | 0.068 |
|  |  |  | 3) | -0.118 | 0.046 | -0.124 | -2.578 | .010 |  |
|  |  |  | 2) | -0.015 | 0.007 | -0.107 | -2.222 | .027 |  |
| Course day 28 | 199 | online |  | 10.992 | 0.747 |  | 14.710 | <.001 |  |
|  |  |  | 2) | -0.036 | 0.010 | -0.247 | -3.527 | <.001 | 0.120 |
|  |  |  | 1) | 0.477 | 0.177 | 0.182 | 2.695 | .008 |  |
|  |  |  | 6) | -0.060 | 0.030 | -0.140 | -2.006 | .046 |  |
|  | 216 | face-to-face |  | 9.456 | 0.645 |  | 14.651 | <.001 |  |
|  |  |  | 1) | 0.500 | 0.164 | 0.204 | 3.049 | .003 | 0.042 |
| Course day 29 | 216 | online |  | 10.536 | 0.777 |  | 13.568 | <.001 |  |
|  |  |  | 1) | 0.764 | 0.168 | 0.305 | 4.537 | <.001 | 0.098 |
|  |  |  | 4) | -0.423 | 0.160 | -0.178 | -2.648 | .009 |  |
|  | 199 | face-to-face |  | 10.402 | 0.681 |  | 15.285 | <.001 |  |
|  |  |  | 6) | -0.081 | 0.028 | -0.200 | -2.903 | .004 | 0.072 |
|  |  |  | 1) | 0.490 | 0.172 | 0.197 | 2.857 | .005 |  |

1) = “I am very well self-organized when it comes to learning.”; 2) = “What is your average travel time in minutes to the Anatomical Institute?”; 3) = “How old are you?”; 4) = “I prefer intensive reviewing and reflecting for learning.”; 5) = “I prefer practical tasks for learning”. 6) = “How many hours per week do you work in addition to your studies to support yourself?”; B = unstandardized Coefficient; SE = standard error; β = standardized coefficient.
